# Fatal, non-fatal burden of cancer in the elderly in China, 2005–2016: a nationwide registry-based study

**DOI:** 10.1186/s12889-023-15686-9

**Published:** 2023-05-12

**Authors:** Zhen-Rong Ma, Kang-Qian Lin, Hong Guo, Kai-Yue Yang, Miao Cao, Xi Song, Huai-Miao Jia, Yun-Hua Hu, Yi-Zhong Yan

**Affiliations:** 1 Department of Preventive Medicine, Medical College, Shihezi University, 129 Bei Er Road, Shihezi, 832002 China; 2 Shihezi Municipal Center for Disease Control and Prevention, Shihezi, 832002 China; 3 Key Laboratory for Prevention and Control of Crucial Emerging Infectious Diseases and Public Health Security of The Xinjiang Production and Construction Corps, Xinjiang Production and Construction Corps, Shihezi, China

**Keywords:** Cancer burden, Elderly, DALY, PYLL, Joinpoint model, China

## Abstract

**Background:**

As populations age, cancer burden becomes increasingly conspicuous. This study quantified the cancer burden of the elderly (≥ 60 years) in China, based on the China Cancer Registry Annual Report to provide epidemiological evidence for cancer prevention and control.

**Methods:**

Data on cancer cases and deaths among the elderly aged ≥ 60 years were collected from the China Cancer Registry Annual Report, 2008–2019. Potential years of life lost (PYLL) and disability-adjusted life years (DALY) were calculated to analyze fatalities and the non-fatal burden. The time trend was analyzed using the Joinpoint model.

**Results:**

From 2005 to 2016, the PYLL rate of cancer in the elderly was stable between 45.34‰ and 47.62‰, but the DALY rate for cancer decreased at an average annual rate of 1.18% (95% CI: 0.84–1.52%). The non-fatal cancer burden in the rural elderly was higher than that of the urban elderly. Lung, gastric, liver, esophageal, and colorectal cancers were the main cancers causing the cancer burden in the elderly, and accounted for 74.3% of DALYs. The DALY rate of lung cancer in females in the 60–64 age group increased (annual percentage change [APC] = 1.14%, 95% CI: 0.10–1.82%). Female breast cancer was one of the top five cancers in the 60–64 age group, with DALY rates that also increased (APC = 2.17%, 95% CI: 1.35–3.01%). With increasing age, the burden of liver cancer decreased, while that of colorectal cancer rose.

**Conclusions:**

From 2005 to 2016, the cancer burden in the elderly in China decreased, mainly reflected in the non-fatal burden. Female breast and liver cancer were a more serious burden in the younger elderly, while colorectal cancer burden was mainly observed in the older elderly.

**Supplementary Information:**

The online version contains supplementary material available at 10.1186/s12889-023-15686-9.

## Introduction

Cancer is a significant barrier to improving life expectancy in every country in the world and will become the leading cause of death in most countries this century [1]. According to the World Health Organization (WHO) estimates in 2019, 112 of the 183 countries had cancer as the first or second leading cause of death before the age of 70 and cancer ranked third or fourth in another 23 countries [2]. Previous studies have shown that China accounts for 23.7% of new cancer cases and 30% of worldwide cancer deaths [3].

Serious population aging may bring about a series of social problems, including that concerning the prevention and control of geriatric diseases. By the end of 2019, the elderly population aged ≥ 60 years in China had reached 253 million, accounting for 18.1% of the population, and China had become a “deep aging society” [4]. Studies have found that cancer was the second leading cause of death in elderly (≥ 60 years) in China from 1990 to 2019, following that of circulatory diseases, and the incidence and mortality of cancer in the elderly were 8.18 times and 13.53 times that of middle-aged and young people, respectively [5, 6]. The prevention and control of cancer in the elderly have become an important public health problem in China.

Therefore, the purpose of this study was to provide a reference for the prevention and control of cancer in the elderly in China through the analysis of the current situation and of the trends in the cancer burden among the elderly from 2005 to 2016, based on the national registration, including fatal and non-fatal burden by calculating the potential years of life lost (PYLL) and disability-adjusted life years (DALY).

## Materials and methods

### Data sources

From the China Cancer Registry Annual Report, 2008–019 [7–18] published by the National Central Cancer Registry of China, the morbidity and mortality information of all cancer patients in the elderly aged ≥ 60 years in China from 2005 to 2016 was selected. These reports included cancer at all anatomical sites (ICD-10: C00–97, D32–33, D42–43, D45, D47) [19].

### Indicators

#### PYLL (potential years of life lost)

The formulas for calculating PYLL and PYLL rate (‰) were as follows [20]:

PYLL = ∑ (L-a_i_) × d_i_.

PYLL rate (‰) = PYLL/n × 1000‰.

In the formula, L is the life expectancy from the sixth national census of China in 2010 [21, 22]; a_i_ is the median of age group i, and the age grouping method in this study is based on the China Cancer Registry Annual Report [7–18], a_i_ for the 60–64 age group was 62, the 65–69 age group was 67, the 70–74 age group was 72, the 75–79 age group was 77, the 80–84 age group was 82, and the over 85 age group was 87; d_i_ is the number of deaths in age group i; n is the total population aged over 60 in the same period.

The difference between L and a_i_ is used to determine whether premature death has occurred. If the difference is greater than 0, premature death is considered to have occurred. The age thresholds for premature death in this study based on life expectancy adjustments are as follows:

The life expectancy is 77 years, and 72 years for males, 77 years for females; the life expectancy of urban residents is 82 years, and 77 years for males, 82 years for females; the life expectancy of rural residents is 72 years, and 72 years for males, 77 years for females.

#### DALY (disability-adjusted life years)

The formulas for calculating DALY and DALY rate (‰) were as follows:

DALY = YLL (years of life lost) + YLD (years lived with disability) [23].

DALY rate (‰) = DALY/n × 1000‰.

In the formula, n is the total population aged over 60 in the same period.

YLL = N × DW × Ce(γα)/(β + γ) ^2^ {e^−(β +γ) (L+α)^ [-(β + γ) (L + α)-1]-e^−( β +γ) α^[-(β + γ) α-1]}

In the formula, N is the number of deaths; DW is disability weight (death is 1); L is the duration of disability or death due to premature death, The value is 80 years for males and 82.5 years for females; γ is the discount rate, the Global Burden of Disease (GBD) standard value is 0.03; C is the normal number of age-weighted schools, and the GBD standard takes a value of 0.1658; α is the age at which death occurred; β is an age-weighted function parameter, and the GBD standard takes a value of 0.04. Use the DALY calculation template provided by the WHO.

YLD = DW × P [24].

In the formula, P is the prevalence; DW is the disability weight, and the DW value is set at 0.288 [25].

### Statistical analysis

#### Differential analysis of cancer burden characteristics

All the analysis was stratified by different regions (urban/rural), sex (male/ female), age group (60–85 years old according to one age group every 5 years old, and 85 years old and older into one group, a total of 6 groups) and years (2005–2016). It was assumed that the PYLL rate of cancer in the population followed the Poisson distribution, and the Poisson regression was fitted by the SAS statistical software Genmod module. The dependent variable was the PYLL rate, and the independent variables were gender, region, and adjusted age group. After adjusting for age, region, and sex, the regional ratio and sex ratio of the PYLL rate in each age group and their 95% CI were calculated, and the linear regression test ratio trend test was performed [26].

#### Trend analysis of cancer burden

Time trend analysis was expressed as an annual percentage change (APC) and its 95% CI, calculated using Joinpoint 4.3.1.0 developed by the American Institute for Cancer Research [27]. If the trend is statistically significant, it is indicated by an “increase” or “decrease”. If not, it is indicated as “stable”.

## Results

### The cancer PYLL in the elderly in China, 2005–2016

#### The cancer PYLL in different sex

From 2005 to 2016, the cancer PYLL of the elderly in China was 13,473,579 person-years, and the PYLL rate was 46.52‰, which was stable over time (*P* > 0.05). The cancer PYLL of the elderly males was 4,406,299 person-years, the PYLL rate was 36.52‰ and decreased at an average annual rate of 1.42% (95%CI: 0.91–1.94%) over time. And that of females was 4,495,040 person-years, the PYLL rate was 30.49‰ and decreased at an average annual rate of 0.64% (95%CI: 0.45–0.83%) over time. The male/female PYLL ratio of rural residents was stable, while the ratios of urban and national residents were decreasing from 2005 to 2016 (all *P* < 0.05). (Tables 1 and 2)

**Table 1:** The cancer PYLL by sex in the elderly in China, 2005–2016

Year	Male		Female		Total	
	PYLL/person-years	PYLL rate/‰	PYLL/person-years	PYLL rate/‰	PYLL/person-years	PYLL rate/‰
2005	93,865	32.60	118,196	32.03	329,279	46.34
2006	104,120	32.96	130,474	32.28	364,694	46.81
2007	105,540	32.89	129,064	31.52	363,288	45.90
2008	122,875	33.51	145,500	30.77	414,734	45.34
2009	161,650	34.57	182,465	31.11	527,817	45.99
2010	247,061	36.60	263,860	31.17	777,284	46.98
2011	303,563	37.35	316,369	31.34	942,259	47.62
2012	416,811	36.97	426,412	30.74	1,276,548	46.83
2013	504,273	37.56	502,840	30.66	1,519,577	47.09
2014	652,152	36.98	646,274	30.21	1,949,453	46.25
2015	760,754	37.01	737,872	29.88	2,254,366	46.26
2016	933,635	36.96	895,714	29.77	2,754,282	46.31
Total	4,406,299	36.52	4,495,040	30.49	13,473,579	46.52
APC/%	1.42		-0.64		0.07	
95%CI	(0.91, 1.94)		(-0.83, -0.45)		(-0.19, 0.32)	
*t*	6.17		-7.50		0.60	
*P*	< 0.001		< 0.001		0.561	

**Note**: PYLL = Potential years of life lost, APC = Annual percentage change, CI = Confidence interval

**Table 2:** The sex ratio (male/female) of cancer PYLL in the elderly in China, 2005–2016

Year	Urban		Rural		Nationwide	
	Sex Ratio	95%CI	Sex Ratio	95%CI	Sex Ratio	95%CI
2005	2.161	(2.100, 2.224)	1.656	(1.630, 1.682)	1.843	(1.818, 1.870)
2006	2.075	(2.014, 2.137)	1.777	(1.752, 1.803)	1.837	(1.813, 1.861)
2007	2.055	(2000, 2.112)	1.722	(1.697, 1.747)	1.797	(1.775, 1.821)
2008	1.964	(1.912, 2.017)	1.731	(1.709, 1.753)	1.779	(1.758, 1.799)
2009	1.883	(1.846, 1.921)	1.725	(1.704, 1.747)	1.772	(1.754, 1.791)
2010	1.942	(1.912, 1.972)	1.786	(1.767, 1.806)	1.839	(1.823, 1.855)
2011	1.953	(1.926, 1.980)	1.776	(1.758, 1.794)	1.842	(1.828, 1.858)
2012	1.933	(1.913, 1.953)	1.717	(1.701, 1.734)	1.821	(1.808, 1.834)
2013	1.885	(1.867, 1.903)	1.712	(1.697, 1.727)	1.794	(1.782, 1.805)
2014	1.844	(1.829, 1.860)	1.700	(1.687, 1.713)	1.767	(1.757, 1.777)
2015	1.828	(1.814, 1.842)	1.677	(1.665, 1.689)	1.751	(1.742, 1.761)
2016	1.835	(1.822, 1.848)	1.714	(1.703, 1.725)	1.771	(1.763, 1.780)
*t*	-7.087		-0.535		-2.253	
*P*	< 0.001		0.598		0.048	

**Note**: CI = Confidence interval

#### The cancer PYLL in different regions

From 2005 to 2016, the cancer PYLL in the elderly in urban areas was 12,175,284 person-years, and the PYLL rate was 66.98‰, which was stable over time (*P* > 0.05). The cancer PYLL in the elderly in rural areas was 6,409,128 person-years. The PYLL rate was 51.30‰ and decreased at an average annual rate of 3.12% (95%CI:2.42–3.82%). At the same time, the urban /rural ratio of cancer PYLL rate was stable from 2005 to 2016 (*P* > 0.05). (Tables 3 and 4)

**Table 3:** The cancer PYLL in the elderly in China, 2005–2016

Year	Urban		Rural		Nationwide	
	PYLL/person-years	PYLL rate/‰	PYLL/person-years	PYLL rate/‰	PYLL/person-years	PYLL rate/‰
2005	409,190	68.41	104,339	64.71	329,279	46.34
2006	491,194	71.03	96,095	66.52	364,694	46.81
2007	459,580	67.85	113,680	65.98	363,288	45.9
2008	531,115	65.15	111,000	63.46	414,734	45.34
2009	603,949	69.17	195,780	55.18	527,817	45.99
2010	840,792	70.29	307,165	53.98	777,284	46.98
2011	941,879	68.79	411,107	55.74	942,259	47.62
2012	1,078,797	67.39	664,408	51.94	1,276,548	46.83
2013	1,188,592	64.41	809,263	51.91	1,519,577	47.09
2014	1,547,779	63.73	1,010,678	49.98	1,949,453	46.25
2015	1,781,927	66.49	1,204,103	49.07	2,254,366	46.26
2016	2,300,489	67.60	1,381,510	48.21	2,754,282	46.31
Total	12,175,284	66.98	6,409,128	51.30	13,473,579	46.52
APC/%	-0.46		-3.12		0.07	
95%CI	(-1.03, 0.11)		(-3.82, -2.42)		(-0.19, 0.32)	
*t*	-1.81		-9.81		0.60	
*P*	0.101		< 0.001		0.561	

**Note**: PYLL = Potential years of life lost, APC = Annual percentage change, CI = Confidence interval

**Table 4:** The regional ratio (urban/rural) of cancer PYLL in the elderly in China, 2005–2016

Year	Male		Female		Total	
	Regional ratio	95%CI	Regional ratio	95%CI	Regional ratio	95%CI
2005	1.133	(1.120, 1.147)	1.250	(1.236, 1.265)	2.120	(2.101, 2.140)
2006	1.176	(1.162, 1.191)	1.202	(1.188, 1.217)	2.147	(2.128, 2.169)
2007	1.094	(1.081, 1.106)	1.197	(1.184, 1.211)	2.046	(2.029, 2.066)
2008	1.172	(1.159, 1.185)	1.231	(1.217, 1.245)	2.047	(2.029, 2.066)
2009	1.405	(1.393, 1.418)	1.381	(1.369, 1.394)	2.467	(2.451, 2.485)
2010	1.463	(1.453, 1.473)	1.434	(1.424, 1.444)	2.522	(2.509, 2.537)
2011	1.433	(1.425, 1.443)	1.302	(1.294, 1.310)	2.382	(2.372, 2.395)
2012	1.495	(1.488, 1.504)	1.385	(1.378, 1.392)	2.492	(2.484, 2.503)
2013	1.506	(1.499, 1.513)	1.363	(1.356, 1.370)	2.368	(2.361, 2.378)
2014	1.573	(1.567, 1.580)	1.382	(1.376, 1.388)	2.419	(2.412, 2.428)
2015	1.619	(1.613, 1.625)	1.430	(1.425, 1.436)	2.490	(2.484, 2.499)
2016	1.727	(1.722, 1.734)	1.419	(1.414, 1.425)	3.095	(3.087, 3.103)
*t*	9.246		4.291		4.258	
*P*	< 0.001		< 0.001		0.002	

**Note**: CI = Confidence interval

### The sequence of cancer PYLL in the elderly in China, 2005–2016

**Fig. 1:**
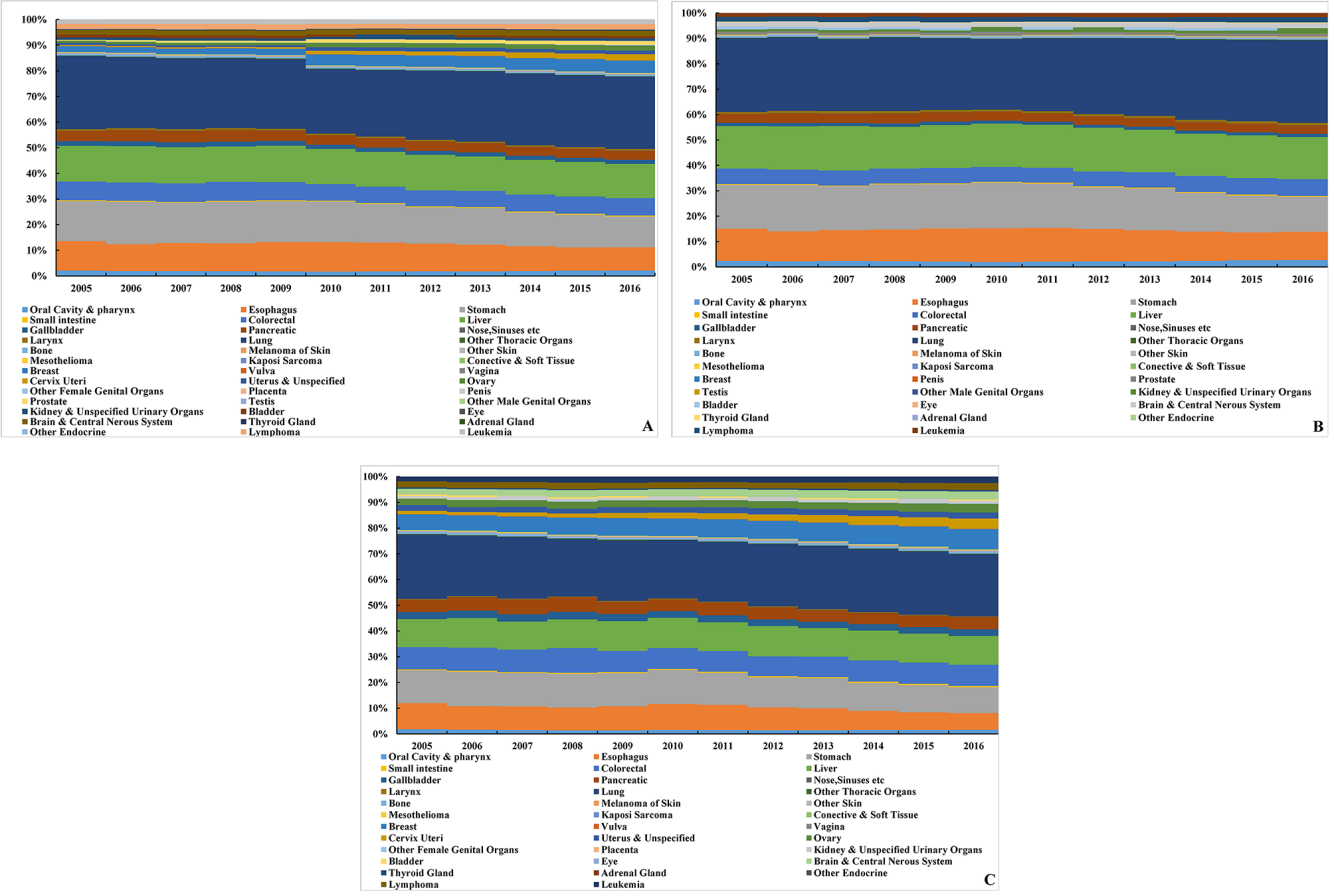
Proportion of PYLL caused by cancers in the elderly in China, 2005–2016 (A. all; B. male; C. female)

Sorted by annual average PYLL, lung cancer caused the most PYLL in the elderly in China from 2005 to 2016, with an average annual PYLL of 317,583 person-years, followed by gastric, liver, esophageal, and colorectal cancer. The burden caused by the above five cancers accounted for 71.3% of the total cancer burden. Cancer with the highest PYLL in males was lung cancer, with an average annual PYLL of 108,990 person-years, followed by liver, gastric, esophageal, and colorectal cancer. It accounted for 79.9% of the total PYLL. Cancer with the highest PYLL in females was also lung cancer, with an average annual PYLL of 88,164 person-years, followed by gastric, liver, colorectal, and esophageal cancer. It accounted for 63.1% of the total PYLL. (Fig. 1)

### Annual trend of the top five cancer PYLL rate in the elderly in China, 2005–2016

**Fig. 2:**
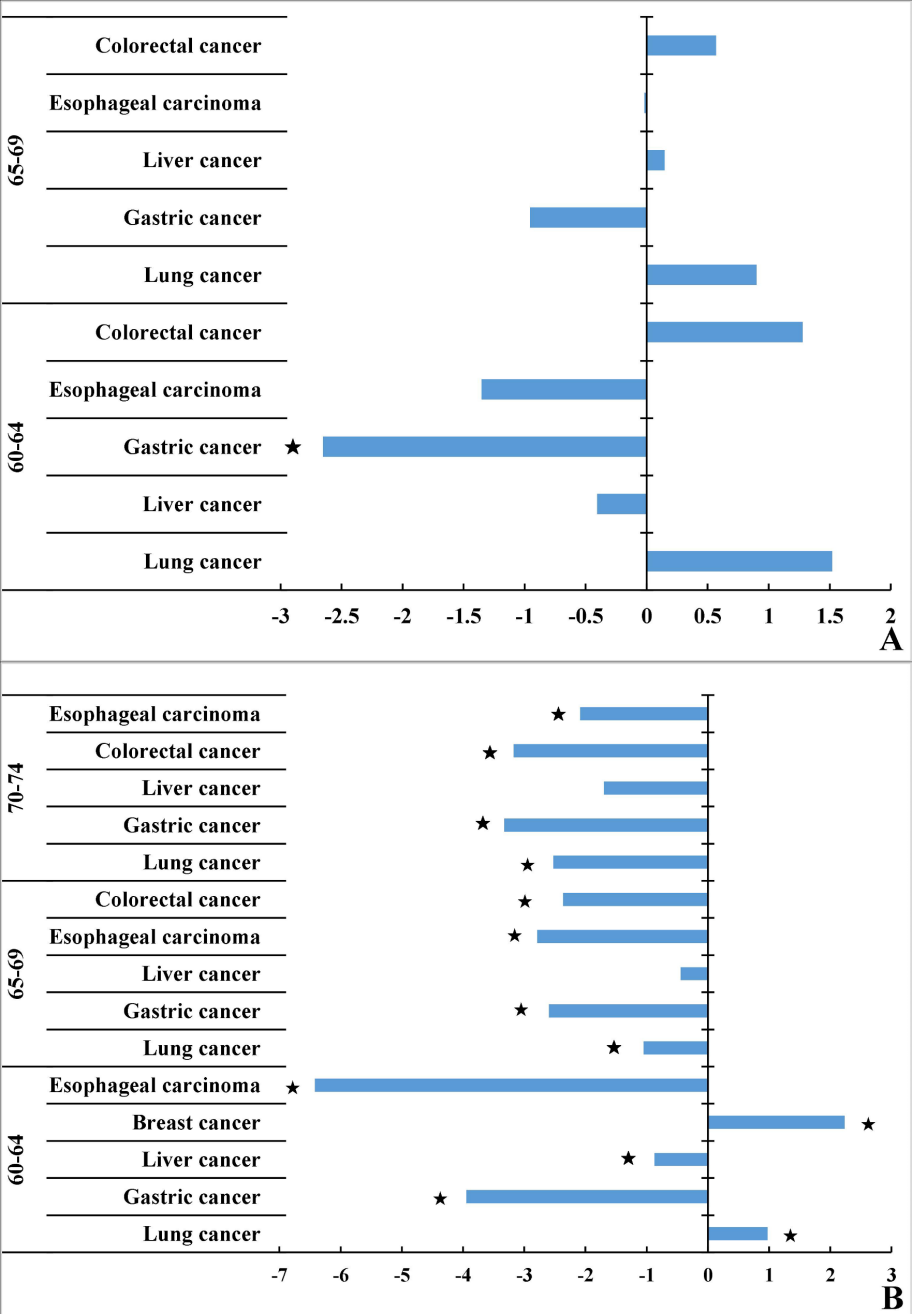
The APC of the top five cancer PYLL rate in the elderly by age, 2005–2016 (A. male; B. female) **Note** ★*P* < 0.05.

From 2005 to 2016, in males, among the top five cancer PYLL rates from different age groups, only the PYLL rate of gastric cancer from the 60–64 age group showed a downward trend (APC = -2.65, *P* < 0.05). In females, the top five cancer PYLL rates in different age groups showed a downward trend over time, except for liver cancer in the 70–74 age group (APC = -0.88%, *P* > 0.05) and 65–69 age group (APC = -0.44%, *P* > 0.05); and it should be noted that in the 60–64 age group, the PYLL rates of lung cancer (APC = 0.98%, *P* < 0.05) and female breast cancer (APC = 2.24%, *P* < 0.05) showed an upward trend from 2005 to 2016. At the same time, only in the 60–64 age group, the PYLL rate of female breast cancer was fourth place, and in other age groups, it was not in the top five. (Fig. 2)

### The cancer DALY in the elderly in China, 2005–2016

#### The cancer DALY in different sex

From 2005 to 2016, the cancer DALY of the elderly in China was 25,921,678 person-years, and the DALY rate was 77.10‰, showing a downward trend at an annual rate of 1.18% (95% CI: 0.84–1.52%). The cancer DALY of the elderly males was 15,744,073 person-years, the DALY rate was 97.15‰, and decreased at an average annual rate of 1.04% (95% CI: 0.71–1.37%). And that of females was 10,177,605 person-years, the DALY rate was 58.44‰, and decreased at an average annual rate of 1.57% (95% CI :1.21–1.92%). (Table 5)

**Table 5:** The cancer DALY in the elderly in China, 2005–2016

Year	Male		Female		Total	
	DALY/person-years	DALY rate/‰	DALY/person-years	DALY rate/‰	DALY/person-years	DALY rate/‰
2005	390,009	102.34	273,156	63.76	663,165	81.92
2006	440,900	105.51	305,527	65.01	746,427	84.07
2007	443,768	103.42	308,397	64.55	752,165	82.94
2008	512,763	102.16	353,109	63.21	865,872	81.64
2009	647,997	101.77	437,343	63.34	1,085,340	81.78
2010	938,247	102.52	624,813	62.65	1,563,060	81.73
2011	1,122,488	102.19	731,550	61.55	1,854,038	81.07
2012	1,502,443	98.79	981,250	59.97	2,483,693	78.67
2013	1,772,214	98.15	1,141,459	58.99	2,913,673	77.89
2014	2,244,867	94.52	1,440,444	56.88	3,685,311	75.09
2015	2,589,112	93.91	1,628,413	55.51	4,217,525	74.11
2016	3,139,265	93.23	1,952,145	54.74	5,091,409	73.43
Total	15,744,073	97.15	10,177,605	58.44	25,921,678	77.10
APC/%	-1.04		-1.57		-1.18	
95%CI	(-1.37, -0.71)		(-1.92, -1.21)		(-1.52, -0.84)	
*t*	-7.017		-9.655		-7.753	
*P*	< 0.001		< 0.001		< 0.001	

**Note**: PYLL = Potential years of life lost, APC = Annual percentage change, CI = Confidence interval

#### The cancer DALY in different regions

From 2005 to 2016, the cancer DALY in the elderly in urban areas was 14,431,915 person-years. The DALY rate was 74.88‰ and decreased at an average annual rate of 0.83% (95% CI: 0.40–1.26%). The cancer DALY in the elderly in rural areas was 11,982,525 person-years. The DALY rate was 81.60‰ and decreased at an average annual rate of 3.38% (95% CI: 2.50–4.24%). (Table 6)

**Table 6:** The cancer DALY in the elderly in China, 2005–2016

Year	Urban		Rural		Nationwide	
	DALY/ person-years	DALY rate/‰	DALY/ person-years	DALY rate/‰	DALY/ person-years	DALY rate/‰
2005	480,099	76.66	187,235	102.18	663,165	81.92
2006	577,362	79.80	173,527	105.59	746,427	84.07
2007	550,718	77.47	205,903	105.08	752,165	82.94
2008	668,869	77.76	201,388	100.48	865,872	81.64
2009	727,971	78.96	359,481	88.70	1,085,340	81.78
2010	1,007,792	79.85	1,003,804	101.77	1,563,060	81.73
2011	1,120,174	77.48	738,065	87.74	1,854,038	81.07
2012	1,287,479	75.88	1,200,346	82.20	2,483,693	78.67
2013	1,456,814	74.40	1,461,106	81.96	2,913,673	77.89
2014	1,878,919	72.70	1,810,431	77.92	3,685,311	75.09
2015	2,059,414	72.06	2,162,066	76.32	4,217,525	74.11
2016	2,616,305	72.16	2,479,172	74.95	5,091,409	73.43
Total	14,431,915	74.88	11,982,525	81.60	25,921,678	77.10
APC%	-0.83		-3.38		-1.18	
95%CI	(-1.26, -0.40)		(-4.24, -2.50)		(-1.52, -0.84)	
*t*	-4.308		-8.483		-7.753	
*P*	0.002		< 0.001		< 0.001	

**Note**: PYLL = Potential years of life lost, APC = Annual percentage change, CI = Confidence interval

#### The sequence of cancer DALY in the elderly in China, 2005–2016

**Fig. 3:**
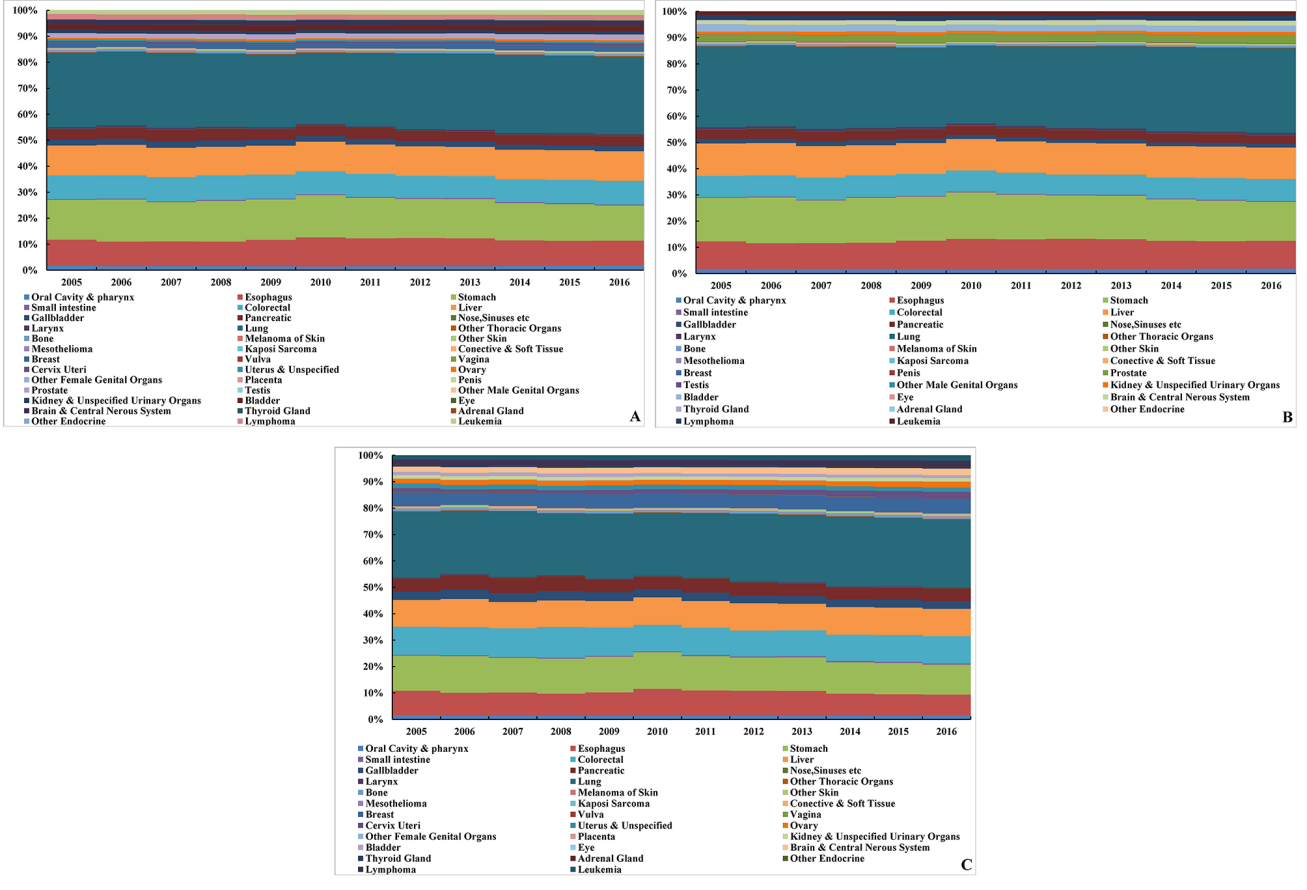
Proportion of DALY caused by cancers in the elderly in China, 2005–2016 (A. all; B. male; C. female)

Sorted by annual average DALY, lung cancer caused the most DALY in the elderly in China from 2005 to 2016, with an average annual DALY of 28,454 person-years, followed by gastric, liver, esophageal, and colorectal cancer. The burden caused by the above five cancers accounted for about 74.3% of the total cancer burden. Cancer with the highest DALY in elderly males was lung cancer, with an average annual DALY of 339,038 person-years, followed by gastric, liver, esophageal, and colorectal cancer. It accounted for 78.4% of the total DALY. Cancer with the highest DALY in elderly females was also lung cancer, with an average annual DALY of 74,832 person-years, followed by gastric, colorectal, liver, and esophageal cancer. It accounted for 67.3% of the total DALY. (Fig. 3)

### Annual trend of the top five cancer DALY rates in the elderly in China, 2005–2016

In males, from 2005 to 2016, the leading cause of cancer burden in all age groups was lung cancer, and the DALY rate was stable over time (*P* > 0.05). The DALY rate of gastric cancer showed a downtown trend over time in the 60–64, 70–74 and ≥ 85 age groups, of which the most obvious trend was in the 60–64 age group (APC_60–64_ = -2.68%, APC_70–74_ = -2.16%, APC_85+_ = -2.40%, all *P* < 0.05). Liver cancer showed a downward trend over time in the 70–74 and the ≥ 85 age group, of which the most obvious trend was in the ≥ 85 age group (APC_70–74_ = -1.50%, APC_85+_ = -2.13%, all *P* < 0.05). The esophageal cancer was stable over time in all age groups (all *P* > 0.05). Colorectal cancer showed a downtown trend over time only in the 70–74 age group (APC_70–74_ = -2.45%, *P* < 0.05).

**Fig. 4:**
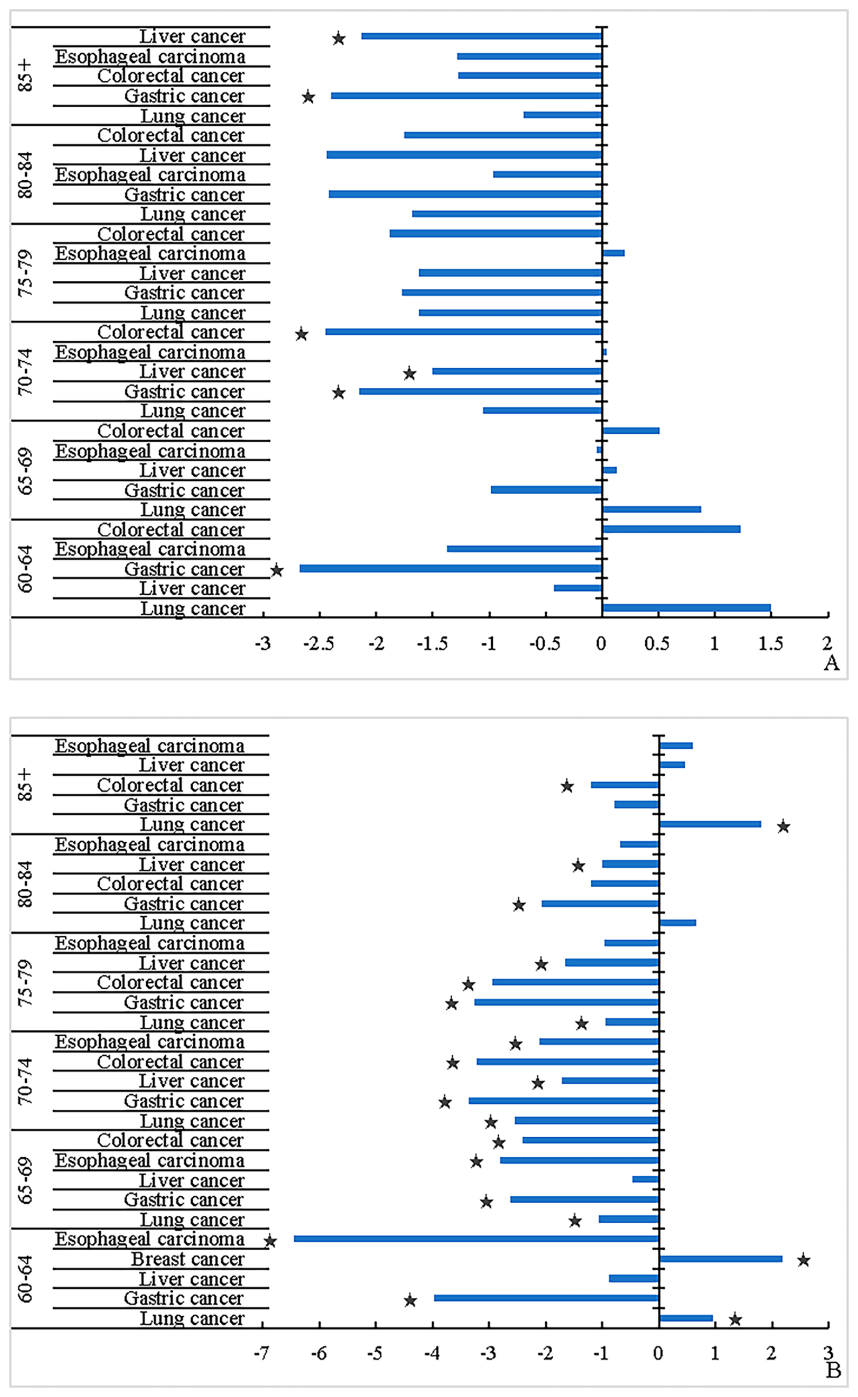
The APC of the top five cancer DALY rates in the elderly by age, 2005–2016 (A. male; B. female) **Note** ★*P* < 0.05

In females, lung cancer was also the leading cause of cancer burden, and the DALY rate of lung cancer showed a downward trend over time in the 70–74 and 75–79 age groups (APC_70–74_ = -2.55%, APC_75–79_ = -0.94%, all *P* < 0.05). Gastric cancer showed a downward trend over time in all age groups under 85, of which the most obvious trend was in the 60–64 age group (APC_60–64_ = -3.97%, APC_65–69_ = -2.62%, APC_70–74_ = -3.36%, APC_80–84_ = -2.06%, all *P* < 0.05). Colorectal cancer showed a downward trend over time in all age groups except for the 80–84 age group (APC_65–69_ = -2.41%, APC_70–74_ = -3.22%, APC_75–79_ = -2.95%, APC_85+_ = -1.20%, all *P* < 0.05). Esophageal cancer showed a downward trend over time in the 60–79 age groups, of which the most obvious was in the 60–64 age group (APC_60–64_ = -6.44%, APC_65–69_ = -2.81%, APC_70–74_ = -2.11%, all *P* < 0.05). And it’s worth noting that, the DALY rate of female breast cancer ranked fourth only in the 60–64 age group and showed an upward trend over time (APC = 2.17%, *P* < 0.05). The DALY rate of lung cancer also showed an upward trend over time in the 60–64 and ≥ 85 age groups (APC_60–64_ = 0.96%, APC_85+_ = 1.80%, all *P* < 0.05). Colorectal cancer ranked fifth in the 65–69 age group, but in all age groups over 75 years old, colorectal cancer ranked third. (Fig. 4)

## Discussion

From 2005 to 2016, the PYLL rate of elderly cancer patients in China fluctuated between 45.34‰ and 47.62‰ and was stable over time; the DALY rate fluctuated between 7.43‰ and 82.94‰, decreasing at an average annual rate of 1.18% (95% CI: 0.84–1.52%). This result may indicate that with economic growth, improvement of medical standards, and advancement in cancer research, Chinese awareness regarding the prevention, diagnosis, and treatment of cancer in the elderly has continuously improved, and certain successes have been achieved in the prevention and treatment of cancer’s non-fatal burden.

From 2005 to 2016, both the PYLL and DALY rates of the elderly males were higher than those of females. The PYLL rate of the elderly males increased with time, while that of females decreased; however, for the DALY rate, it was reduced for both males and females, but the rate of the reduction in females was higher than that of males (APC_male_ = -1.42%, APC_female_ = -1.57%). Therefore, the prevention and treatment effect of the cancer burden in elderly males was worse than that of females, especially with respect to fatal cancer burden. Zhang S et al. [28] have shown that males are more likely to be exposed to risk factors, such as smoking, harmful drinking, exposure to dust, and physical inactivity, resulting in a higher incidence of cancer. To effectively reduce the cancer burden in the elderly in China, the prevention and treatment of cancers in elderly males should be the prime focus. We should mobilize relevant departments to educate the public about health, emphasizing the cancer burden in elderly males. To reduce the occurrence and development of cancer requires identifying and controlling the risk factors, such as smoking, harmful drinking, and exposure to dust, but these lifestyle factors need to be initiated early in life to prevent the development of cancer. At present, WHO’s MPOWER package has been implemented in China, but some projects were not enough [29]. In the future, the coverage of health warnings in China needs to be expanded, the tobacco tax should be increased, and smoke-free laws should be improved. The study showed that the non-price measures would reduce the smoking rate in China by 2.7% [29]. The implementation of the above measures may play an important role in optimizing the allocation of health resources and focusing on reducing the cancer burden among the elderly males in China.

The PYLL rate of elderly cancer patients in China was generally stable over time, while the DALY rate was decreasing. Meanwhile, the PYLL rate of the elderly in rural areas was much lower than that in urban areas, but the DALY rate was higher. Our results suggested that the cancer burden of the elderly rural was higher than that of the urban elderly, and the proportion of the non-fatal burden was also higher. In our previous study [30], we found that from 2005 to 2016, the cancer incidence among elderly people in rural areas was significantly higher than that in urban areas. At the same time, compared with urban areas, cancer patients in rural China may have lower disposable income, poor medical insurance treatment, relatively scarce available medical resources, less available medical services, and poor medical awareness [31–33], which may lead to them being more likely to miss the best opportunity for treatment. Therefore, rural patients are more likely to have a poor prognosis than urban cancer patients and may have a greater non-fatal burden. Narrowing the wealth gap between rural and urban areas, allocating more medical resources in rural areas, improving the medical awareness of rural patients, and implementing early cancer prevention and control strategies may have a significant impact on effectively reducing the cancer burden of the elderly in China. For example, screening is known to be an effective means of early cancer control. The Japanese government has its citizens regularly screened at appropriate ages for gastric, breast, lung, colorectal and cervical cancer, which has resulted in a five-year survival rate of 68.6%, the highest in the world [34]. At the same time, Britain’s breast cancer mortality rate fell to 55.1% between 1990 and 2013 compared with the 1980s, when breast cancer screening was not implemented, and after the introduction of colorectal cancer screening in 2006, the mortality rate of colorectal cancer in men and women decreased to 88.2% and 92.1%, respectively [35, 36]. Although pilot screening trials for these cancers were conducted in a number of medium-sized and above cities in 2012 in China, even the most extensive coverage for breast cancer screening was less than 50% as of 2019 [37]. So, these screening efforts should be extended to all regions of China, not just some urban areas, to reduce the burden of cancer.

From the perspective of the order of cancer burden and the changing trend in the burden of important cancers, lung, gastric, liver, esophageal, and colorectal cancers were more health-threatening to the elderly in China, of which the PYLL and DALY of elderly males and females ranked first in lung cancer. It can be seen that lung cancer still causes the highest cancer burden in the elderly in China. Regarding PYLL and DALY rates of several other cancers with a high cancer burden, for elderly males, the cancers showed a downward trend or stabilized state over time. Among elderly females, in addition to the PYLL rate of lung cancer in the 60–64 age group, the lung cancer DALY rate in the 60–64 age group and the ≥ 85 age group and the PYLL and DALY rates of female breast cancer in the 60–64 age group increased over time. The remaining cancers showed a downward trend or stabilized over time. Gastric cancer showed a downward trend or stabilized in all age groups in both males and females. In recent years, China has achieved good results in the prevention and treatment of gastric cancer. Previous study have shown excellent diagnosis and treatment of early gastric cancer, the 5-year survival rate has been shown to reach more than 90%, the overall prevention and treatment of gastric cancer is better, and the value of improving early screening of gastric cancer is better appreciated and has increased [38]. To effectively reduce the burden of cancer in the elderly in China, improving early screening of gastric cancer can achieve great benefits. At the same time, for both elderly males and females, with age, the order of the burden of disease caused by liver cancer decreases, which may be related to the more advanced stage of primary liver cancer at a younger age [39]. Britain and America have lower rates of gastric and liver cancer than China, maybe thanks to better control of cancer-related infections such as H. pylori and HBV [40, 41]. Therefore, we should strengthen the control of these cancer-associated infections. As the highest risk factor for gastric cancer, the control of H. pylori should be emphasized. The media should educate the public about the dangers of H. pylori and advocate for regular non-invasive testing for H. pylori (including ^13^ C-urea breath test, H. pylori fecal antigen test and serological test), and patients with gastritis should be additionally advised to undergo endoscopy. In addition, the Chinese government added HBV vaccination to the planned immunization of newborns in 1992, and provided free vaccination services for newborns in 2002 [42], this may reduce the burden of liver cancer among the elderly in China in the future.

It is worth noting that in the elderly female population, colorectal cancer has increased in the ranking of loss of healthy life caused by cancers with age. In the PYLL ranking, colorectal cancer was not in the top five ranks in the 60–64 age group but ranked fourth in the 70–74 age group. In the DALY ranking, colorectal cancer ranked fifth in the 65–69 age group, and in all age groups over 75 years old, colorectal cancer ranked third as a cancer that affects the life and health of elderly females. Previous literature has shown that Sirota monotherapy is effective in improving the quality of life of elderly patients with advanced colorectal cancer [43, 44]. Moreover, the PYLL and DALY top five-digit cancers in all age groups were the same as the overall top five-digit cancers, but in females aged 60–64 years, both PYLL and DALY for female breast cancer were in fourth place, and both PYLL and DALY rates increased over time. Chen W et al. [45] showed that the prognosis of female breast cancer was better and the survival rate was higher. The prevention and treatment of breast cancer in females under 65 years old and colorectal cancer in females over 65 years old should be the focus of public health work. The increased incidence of breast and colorectal cancer may be related to the increased intake of processed meat, obesity and sedentary lifestyles after economic development [35]. In order to reduce their cancer burden, in addition to increasing their screening sites across the country and strengthening their early prevention, the media should also promote proper lifestyle behaviors, such as reducing the intake of processed meat, promoting exercise and weight control.

## Conclusion

In summary, encouraging results have been achieved in preventing and treating cancer in the elderly in China, especially in terms of the burden of non-fatal cancers. However, the cancer burden in rural elderly patients was still higher than that in urban elderly patients, and the non-fatal burden of cancer in rural elderly patients was still more serious. The cancer burden in elderly male patients was higher than that in elderly female patients. In the future, it is necessary to focus on the prevention and treatment of cancers with high burdens such as lung cancer, gastric cancer, esophageal cancer, liver and colorectal cancer (in the elderly population of high age), and breast cancer (in the elderly female of low age). Considering the contribution of these cancers to the burden on the elderly, cancer prevention strategies and policies should be formulated and continuously optimized to make them more targeted and precise. Additionally, the hardware facilities and professional level of staff in primary medical institutions are the keys to the detection of early cancer cases. Ensuring the advanced and perfect hardware equipment of primary medical institutions and strengthening the screening and diagnosis training of primary medical staff are beneficial to the effective prevention and treatment of cancer, and have significance for effectively reducing the cancer burden of the elderly.

## Electronic supplementary material

Below is the link to the electronic supplementary material.

Supplementary Material 1

## Data Availability

All data generated or analyzed during this study are included in this published article and its supplementary information files.

## Abbreviations

APC: Annual percentage change
CI: Confidence interval
DALY: Disability-adjusted life years
GBD: Global Burden of Disease
PYLL: Potential years of life lost
YLD: Years lived with disability
YLL: Years of life lost.
